# MiMiC: Multiscale Modeling in Computational Chemistry

**DOI:** 10.3389/fmolb.2020.00045

**Published:** 2020-03-20

**Authors:** Viacheslav Bolnykh, Jógvan Magnus Haugaard Olsen, Simone Meloni, Martin P. Bircher, Emiliano Ippoliti, Paolo Carloni, Ursula Rothlisberger

**Affiliations:** ^1^Laboratory of Computational Chemistry and Biochemistry, École Polytechnique Fédérale de Lausanne, Lausanne, Switzerland; ^2^Hylleraas Centre for Quantum Molecular Sciences, Department of Chemistry, UiT the Arctic University of Norway, Tromsø, Norway; ^3^Dipartimento di Scienze Chimiche e Farmaceutiche, Università degli Studi di Ferrara, Ferrara, Italy; ^4^Computational and Soft Matter Physics, University of Vienna, Vienna, Austria; ^5^Computational Biomedicine, Institute for Advanced Simulation (IAS-5) and Institute of Neuroscience and Medicine (INM-9), Molecular Neuroscience and Neuroimaging, Institute of Neuroscience and Medicine (JARA INM-11), Forschungszentrum Jülich, Jülich, Germany; ^6^Department of Physics and Universitätsklinikum Aachen, RWTH Aachen University, Aachen, Germany

**Keywords:** molecular dynamics, QM/MM, DFT, HPC, multiscale simulations, computational chemistry

## 1. Introduction

Hybrid quantum mechanics/molecular mechanics (QM/MM) approaches are commonly used methods for investigating a plethora of chemical, biochemical, and biophysical processes that require explicit treatment of the electronic degrees of freedom when the system is too big to be entirely treated by QM methods alone (Warshel and Levitt, 1976; Senn and Thiel, 2009; Adhireksan et al., 2014; Campomanes et al., 2014, 2015; Brunk and Rothlisberger, 2015; Genna et al., 2016; Li et al., 2017; Cupellini et al., 2018; Loco et al., 2018; Morzan et al., 2018). It is often the method of choice for computational investigations of systems with more than a few thousand atoms (which is commonly the case for biological systems). In QM/MM, the system is split into two parts: a smaller part that is treated at the QM level of theory, whereas the remainder is described at the MM level, which is a computationally more expedient description. In this way, local electronic effects can be captured with the accuracy of a first-principles method, while at the same time explicitly including the effects of the environment at a reasonable computational cost. Current QM/MM implementations have roughly followed either of two strategies: (1) tight integration of QM and MM modules in a single software package or (2) loose coupling of separate QM and MM codes. Strategy (1) generally profits from computational efficiency due to the ability to pass data between the submodules directly (via function calls) but suffers from limited flexibility, since the available choice of methods is often restricted and extensions to different programs may require formidable programming efforts. In contrast, strategy (2), which is typically implemented resorting to data exchange between QM and MM codes *via* file input and output, enables high flexibility but penalizes efficiency because of increased communication overhead. However, with the field rapidly growing, new simulation paradigms and approaches might quickly emerge, clearly favoring strategy (2) over (1). In the following, we show that flexibility does not necessarily come at the expense of a high computation (or communication) overhead by presenting the recently developed MiMiC framework (Bolnykh et al., 2019; Olsen et al., 2019) that combines the capability of performing fast and efficient multiscale molecular dynamics (MD) simulations with facile support for flexible extensions. These objectives are achieved by applying (2) with an efficient method to exchange data among the coupled software packages. In practice, MiMiC implements a multiple program-multiple data (MPMD) paradigm through a message passing interface (MPI)-based communication library, which allows the entities collaborating within MiMiC to exchange data efficiently. Overall, MiMiC represents a highly modular and general multiscale simulation framework that enables the combination of multiple resolutions and methods for different parts of a system, while retaining high computational efficiency. Moreover, MiMiC was designed to have a flexible architecture enabling multiple resolutions, implementation of different types of coupling (e.g., QM/QM, QM/QM/MM, etc.), and to straightforwardly incorporate emerging—and future—methods and software packages in the field of computational chemistry. This flexibility is of utmost importance in the light of the rapid development of computational methods enabling researchers to tackle complex scientific problems with more and more degrees of freedom that require the incorporation of multiple space and time resolution scales on the one hand, and the rapid advent of new computational approaches on the other hand.

## 2. MiMiC Architecture

### 2.1. Model

MiMiC implements a generalized version of the fully Hamiltonian electrostatic embedding scheme introduced in Laio et al. (2002). The key quantity is the electrostatic QM/MM coupling energy term:

(1) $$\begin{array}{l} E^{\text{QM}/\text{MM}}=\overset{N^{\text{MM}}}{\underset{i}{\sum }}q_{i}^{\text{MM}}\int \text{d}\text{r}\rho^{\text{QM}}\left(\text{r}\right)\frac{r_{c,i}^{4}-|{\text{R}}_{i}-\text{r}{|}^{4}}{r_{c,i}^{5}-|{\text{R}}_{i}-\text{r}{|}^{5}} \end{array}$$

where *N*^MM^ is the total number of MM atoms, $q_{i}^{\text{MM}}$ and *r*_*c, i*_ are the partial charge and the covalent radius of the *i*-th MM atom, respectively, while **R**_*i*_ is its coordinate and ρ^QM^(**r**) is the electron density in point **r**. This form of the electrostatic QM/MM coupling term modifies the Coulomb interaction at short range, thus avoiding electron spill-out (Laio et al., 2002). It is worth remarking that the QM/MM term is responsible for the polarization of the electronic density due to MM atoms and, thus, models the effects of the environment on the properties of the chemically active subdomain.

The straightforward implementation of such a term is rather costly to compute, in particular for systems with large MM regions. Therefore, a hierarchical electrostatic embedding approach (Laio et al., 2002) is used in order to mitigate the high computational cost of a direct evaluation. Within this hierarchical scheme the QM/MM electrostatic interactions are divided into two groups depending on the distance (commonly referred to as the cutoff distance) of MM atoms from the QM subsystem. In the vicinity of the QM part the interaction is computed using Equation (1), whereas more distant atoms are coupled via a multipole expansion of the electrostatic potential of the QM charge distribution. We have extended the original scheme with an open-ended multipole expansion allowing the user to choose the order at which the expansion is truncated. This allows (i) higher accuracy in the calculation of the electrostatic QM/MM interactions, at a negligibly higher computational cost and (ii) reduction of the cutoff distance, thus further lowering the computational cost (Olsen et al., 2019).

An official release of MiMiC will be published under the open-source GPLv3+ license in 2020.

### 2.2. Implementation

MiMiC is a loosely-coupled MPMD multiscale simulation framework. Within this approach, both QM and MM codes run simultaneously with computational resources being allocated separately to either entity. Moreover, while enabling efficient communication, such an approach avoids tight integration of MiMiC into either code, which would incur a high implementation and maintenance effort. This enables the construction of a highly modular and efficient multiscale simulation framework capable of coupling virtually any set of simulation codes with the potential for extending it further to enable the support of alternative levels of theory such as a different QM method, coarse-grained approaches, or approaches based on artificial intelligence (Behler and Parrinello, 2007; Christensen et al., 2019; Singraber et al., 2019). In the present implementation, CPMD 4.3 (Hutter et al., 2018) computes the QM contributions, while GROMACS 2019 (Spoel et al., 2005; Abraham et al., 2015, 2019) computes the classical interactions within the MM subsystem as well as all bonded and Lennard-Jones interactions crossing the QM/MM interface. The electrostatic QM/MM interactions are computed by MiMiC. Finally, CPMD integrates the equations of motion.

**Figure 1:**
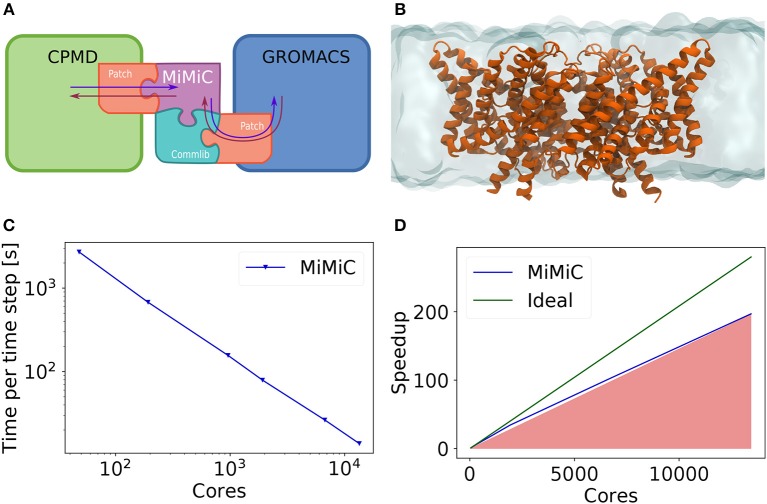
**(A)** Schematic representation of a MiMiC-based QM/MM framework. Patches both for QM and MM codes are required in order to enable the QM/MM workflow. MiMiC then handles all data interactions (depicted as arrows) and routes the relevant information via the communication library (Commlib). **(B)** The test system used for our benchmark consisting of a membrane protein embedded in a lipid bilayer. **(C)** Measured wall-time per time step of a BO MD in a MiMiC QM/MM with B3LYP simulation for the system shown in **(B)**. **(D)** Strong scaling benchmark of a MiMiC QM/MM MD simulation for the system shown in **(B)**.

The structure of a QM/MM implementation using the MiMiC framework is shown in Figure 1A. The use of a plane wave-based code to handle the QM subsystem ensures highly efficient scaling performance, while GROMACS guarantees expedient MM computations.

The workflow of a QM/MM MD simulation using MiMiC follows closely the workflow of a typical MD simulation in CPMD. At the beginning of each time step, MiMiC collects atomic coordinates from CPMD and dispatches them to GROMACS, which then computes MM forces and energies. While this is done, CPMD computes QM contributions and MiMiC computes the electrostatic QM/MM interaction terms. MiMiC adds up all force contributions and provides them to CPMD, which uses them to propagate atomic positions according to the selected ensemble and imposing the necessary constraints.

The calculation of the QM/MM interactions of Equation (1) can be parallelized by distributing MM atoms and points of the mesh discretizing the QM domain of integration. Extreme scalability is achieved parallelizing over both degrees of freedom through a multi-layered hybrid distributed- and shared-memory parallelization strategy. At the top layer, all MPI tasks are divided into groups, each receiving a subset of MM atoms. Then, at a lower level, the mesh discretizing the QM subspace is split into a set of 2D slabs along the *X* dimension. Each of the MPI tasks belonging to each group receives a subset of these slabs to compute the corresponding part of the integral in Equation (1) (and other analogous terms). Finally, at the lowest level, the shared-memory simultaneous multi-threading (SMT) approach (based on OpenMP) is employed in order to further extend the scalability limit. At this level, each of the slabs is divided into a set of 1D "pencils," which are then attributed to the threads associated with a particular MPI task.

Using this multi-layered parallelization scheme, we have demonstrated efficient scalability using over ten thousand cores in a single QM/MM MD simulation while maintaining an overall parallel efficiency above 75% for a system containing a large Cl^−^/H^+^ antiporter protein embedded in a lipid membrane bilayer (Figure 1B) solvated in water. In this system, 19 atoms out of a total of 150,925 atoms were treated at the QM level. The size of the whole system was 126.9 x 126.8 x 99.3 Å^3^, and the size of the cubic QM box was 17.7 x 17.7 x 17.7 Å^3^. We used a plane wave cutoff of 90 Ry, which corresponds to a real-space mesh with 240 points along each dimension. Benchmarks were performed using Troullier–Martins pseudopotentials (Troullier and Martins, 1991). The average wall time of a single MD time step is around 13 s (Bolnykh et al., 2019) when computationally demanding hybrid exchange–correlation functionals, such as B3LYP (Becke, 1988, 1993; Lee et al., 1988), are employed. This enables nanosecond-scale QM/MM MD simulations to be performed, which in turn allows one to obtain converged free energy calculations of biological systems if enough computational resources are available. Some representative scaling benchmark results are shown in Figures 1C,D. We expect similar extreme scalability for systems characterized by QM domains of similar size.

## 3. Conclusion

We have given a short introduction to the recently developed MiMiC framework as a highly flexible and extremely powerful multiscale modeling software solution capable of delivering unprecedented levels of scaling performance. The efficiency of the framework is ensured by using a well-established and extensively validated electrostatic embedding scheme while flexibility and modularity is achieved via an efficient loosely coupled MPMD architecture. Finally, extreme scalability is attained through a multi-layered parallelization strategy.

## Author Contributions

All authors listed have made a substantial, direct and intellectual contribution to the work, and approved it for publication.

### Conflict of Interest

The authors declare that the research was conducted in the absence of any commercial or financial relationships that could be construed as a potential conflict of interest.
